# A pilot study of the shapes of ablation lesions in the canine prostate by laser, radiofrequency and microwave and their clinical significance

**DOI:** 10.1371/journal.pone.0223229

**Published:** 2020-04-09

**Authors:** Ruiqing Liu, Shaobo Duan, Huicun Cao, Guangshao Cao, Zhiyang Chang, Ye Zhang, Yaqiong Li, Yuejin Wu, Luwen Liu, Lianzhong Zhang

**Affiliations:** 1 Department of Interventional Therapy, People’s Hospital of Zhengzhou University, Henan Provincial People’s Hospital, Zhengzhou, Henan, China; 2 Ultrasonic Multimodal Molecular Imaging Laboratory, Henan Provincial People’s Hospital, People’s Hospital of Zhengzhou University, Henan University People’s Hospital, Zhengzhou, Henan, China; 3 Department of Ultrasound, Henan Provincial People’s Hospital, People’s Hospital of Zhengzhou University, Henan University People’s Hospital, Zhengzhou, Henan, China; University Medical Center Utrecht, NETHERLANDS

## Abstract

To explore the shape characteristics of ablation lesions created via laser ablation (LA), radiofrequency ablation (RFA) and microwave ablation (MWA) in canine prostates and the clinical significance of these characteristics, six adult male beagles were randomly assigned to the LA, RFA, and MWA groups. These ablations were performed with common parameters applied in clinical practice (LA, 3 W/1200 J; RFA and MWA, 30 W/120 s). One ablation lesion was created in each lobe of the prostate via the ablation technique, resulting in a total of twelve ablation lesions. Transrectal ultrasound (TRUS) was used as guidance during puncture and to monitor changes in the ablation lesions. Finally, the ablation efficacy was assessed using transrectal contrast-enhanced ultrasonography (CEUS), and the transverse diameter (TRD), anteroposterior diameter (APD) and longitudinal diameter (LD) of each ablation lesion were measured. The volume (V) and the ratio (R) value were calculated. R reflects the shape characteristic of the ablation lesion (the R value close to 1.0 indicates a more spherical shape). The R values of the ablation lesions were 0.89 ± 0.02, 0.72 ± 0.01, and 0.65 ± 0.03 for RFA, MWA and LA, respectively, and they were significantly different (*P* = 0.027). The volumes of the ablation lesions were 2.17 ± 0.10 ml, 1.51 ± 0.20 ml, and 0.79 ± 0.07 ml for MWA, LA and RFA, respectively, and they were also significantly different (*P* = 0.001). The three abovementioned thermal ablation techniques with common parameters in clinical practice can be used for ablation in the prostate. The shapes and volumes of the ablation lesions of the three techniques were varied: The RFA-created lesions had the lowest volumes and were more spherical in shape, demonstrating that RFA could be used for the treatment of relatively small lesions or tumours adjacent to vital organs. The MWA lesions had the largest size with a spherical shape, which could be advantageous for the ablation of tumours with relatively large sizes. The sizes of the ablation lesions created via LA were between those of RFA and MWA but presented more oval in shape, suggesting that this method is highly appropriate for the ablation of benign prostatic hyperplasia (BPH).

## Introduction

Benign prostatic hyperplasia (BPH), a condition often associated with lower urinary tract symptoms (LUTS), commonly occurs in middle-aged and older men [1]. Drug therapies and surgery have been used as common treatments; however, both approaches are accompanied by numerous side effects [2]. Epidemiological studies have shown that the incidence and mortality of prostate cancer (PCa) are increasing [3]. The conventional therapeutic strategies of PCa, including operation, radiotherapy and endocrine therapy, may affect sexual function, urination or defecation [4,5].

Minimally invasive local therapy possesses several dominant advantages, including minimal invasiveness, repeatability, and low incidence of side effects. Therefore, this method has received increasing attention and has been extensively applied in clinical practice [6,7]. Laser ablation (LA), radiofrequency ablation (RFA) and microwave ablation (MWA) are local therapy of thermal ablation techniques that can cause high temperatures and irreversible damage within tissues [8,9]. LA has already been performed in a few countries as a local therapy for prostate diseases, and its effectiveness and safety profiles have been demonstrated [10,11]. RFA and MWA have been validated in animal experiments, and pathological examinations demonstrated that RFA and MWA can induce coagulative necrosis. Furthermore, high-power MWA (45 W/180 s) can penetrate through the prostate, suggesting that appropriate parameters should be taken into consideration in practice [12,13].

To date, how to choose among the three thermal ablation technologies for the treatment of BPH and PCa has not been clearly reported. In the present study, animal experiments were conducted to preliminarily explore the shape and volume characteristics of ablation lesions in the canine prostates using the three techniques with parameters corresponding to those commonly used in clinical practice. The aim is to provide a basis for selecting an optimal thermal ablation therapy for prostate diseases.

## Materials and methods

### Animals

The subjects were six male adult beagles with a mating history from Dilepu Biomedical Co., Ltd. (Xian, China). The animals were weighed and randomly assigned by a random number table to the LA, RFA or MWA groups. The mean age was 5.2 ± 0.8 years old, and the mean body weight was 14.2 ± 2.2 kg. The canines were housed in the Centre for Drug Safety Evaluation of Zhengzhou University (Zhengzhou, China) individually in stainless steel cages (L 1000 mm × W 1000 mm × H 2100 mm) before and after ablation. The environment in which the canines were maintained consisted of a temperature of 23 ± 3°C, 55 ± 15% relative humidity, ventilation 10~20 times/h, and a 12 h light and dark cycle (lights on at 8 a.m. and lights off at 8 p.m.) with 150~300 lux of luminous intensity. Each canine was provided a daily ration of 300 g of solid food (Beijing Keao Xieli Feed Co., Ltd.), and any uneaten food was collected the following morning. Water was disinfected by an ultraviolet sterilizer and ultrafiltration and made available ad libitum via an automatic water supplier.

The study protocol was approved by the Ethics Committee of the Centre for Drug Safety Evaluation of Zhengzhou University (Zhengzhou, China, Protocol Number: ZZUCDSER012). The ethics committee of this organization consists of animal welfare experts. All operation was performed under anaesthesia, and all efforts were made to minimize suffering and discomfort.

### Equipment

An ultrasound system (BK3000; B-K Medical Systems, Inc., Peabody, MA, USA) equipped with a transrectal probe and contrast-tuned imaging (CnTI) was used in the current study.

For LA, an Echolaser X4 (Esaote SpA, Genoa, Italy) with an Nd: YAG laser emission source (wavelength 1064 nm) was used. The laser fibre (300 μm in diameter and 20 cm in length) was placed through a 21 G guidance needle until 1.0 cm of the nude fibre tip was in direct contact with prostate gland tissue, and then the fibre was connected to a continuous wave emission source. For RFA, an LDRF-120S RFA instrument (Lead Electron Corp., Mianyang, China) and LDDJs3-0080100B radiofrequency electrodes were used, with a working tip of 1 cm. For MWA, an ECO-100E1 (MWA, Ecosystems Ltd., Leigh, UK) at a frequency of 2450 MHz and maximal output power of 100 W was utilized. The antenna (ECO-100AL2) had a 1.4 mm outer diameter and was 15 cm in length. Cold circulation inside the electrode and antenna was used to ensure the needle trunk temperature was maintained below 10 °C.

### Contrast agents and anaesthetics

SonoVue (Bracco Group, Milan, Italy) was used as a contrast agent for ultrasound examinations in the present study. The contrast agent was “bolus-like” injected at a dose of 0.04 ml/kg [14]. Pentobarbital sodium (Sinopharm Chemical Reagent Co., Ltd., Shanghai, China), Zoletil 50 (Virbac Inc., Carros, France) and sevoflurane (Shanghai Hengrui Pharmaceutical Co., LTD, Shanghai, China) were used as the sedatives and anaesthetics in this study. Glycerine (Hubei Ketian Pharmaceutical Co. LTD, Hubei, China) was used to perform the enemas.

### Methods

#### Preoperative preparation

After the canines were fasted for 12 h, the skin of the abdomen and perineum region was prepared, and a clean enema was performed. An indwelling needle was inserted into the forearm vein. Anaesthesia was induced by intramuscular injection of pentobarbital sodium (15 mg/kg) and Zoletil 50 (2 mg/kg). The canine was then endotracheally intubated and connected to a respirator (Hamilton Medical AG, USA). Anaesthesia was maintained with of 2% ~ 3% aerosolized sevoflurane in oxygen during the procedure. The tidal volume was set to 10 ml/kg, and the respiratory rate was maintained at 16 breaths/min. After induction of anaesthesia, the canines were placed in the supine position and warmed with a blanket, and the perineal area was local disinfected and draped.

#### Ablation

**Fig 1:**
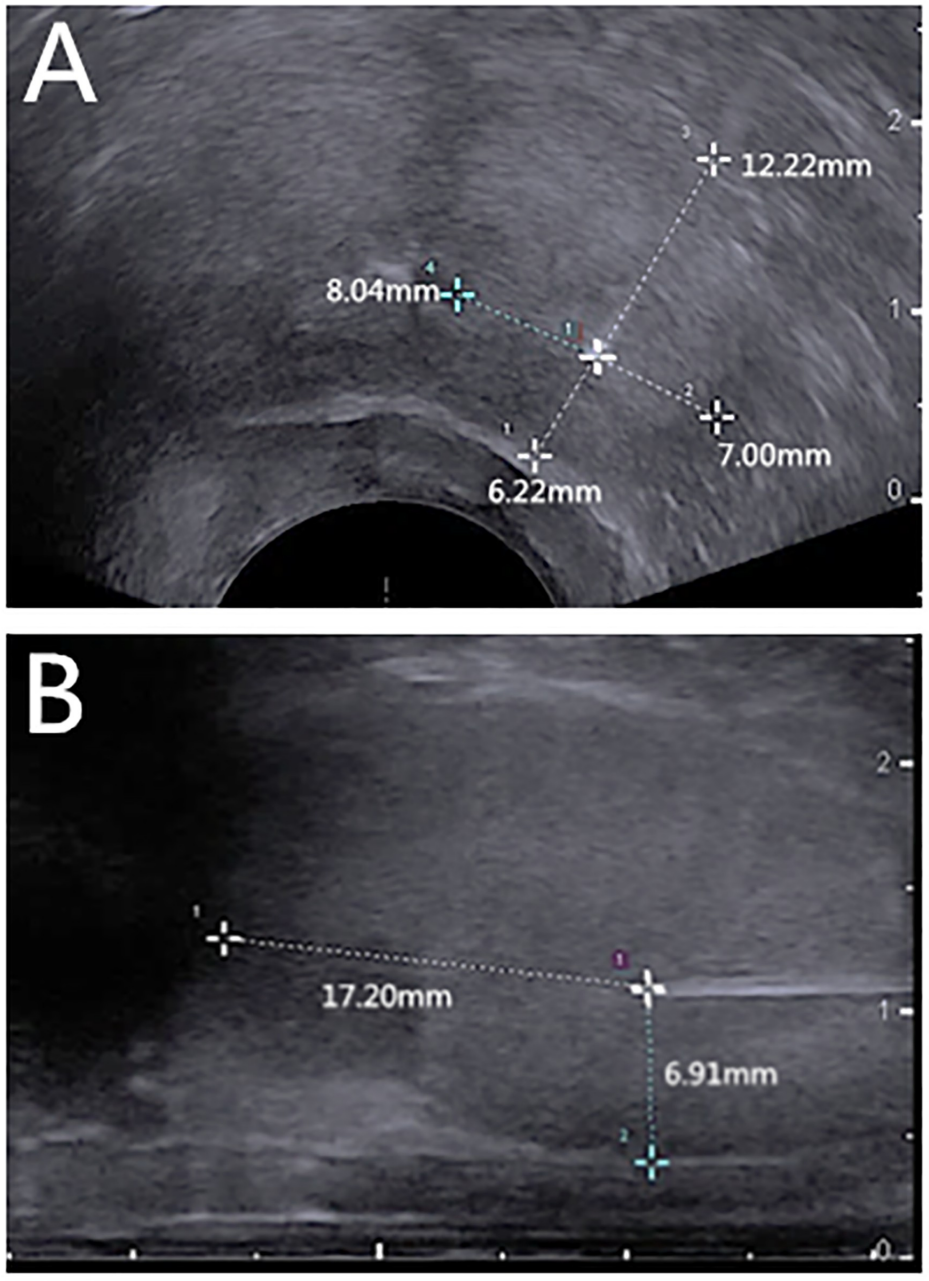
Distances from the tip of the laser fibre to the outer wall of the urethra, the rectum wall and the bladder floor on transrectal ultrasound images (A, transverse section; B, longitudinal section).

The ablation in all the canines was performed by a physician with over five years of ablation experience. Transrectal ultrasound (TRUS) examination was initially conducted for gross scanning of the prostate. The size of the prostate was measured, and then the puncture site in the perineum region was selected. Afterward, the energy transmitter was inserted into the prostate tissue through the perineum under TRUS guidance. The distance from the tip of the transmitter to the outer wall of the urethra, the rectum wall and the bladder floor was at least 6 mm, while the distance from the tip of the laser fibre to the bladder floor was at least 15 mm (Fig 1). The ablation process was continuously monitored via TRUS. For each canine, ablation was performed twice to generate two ablation lesions on two lobes of the prostate according to previously reported parameters used in the thyroid gland [13,15,16]. There were twelve ablation lesions in total, with four lesions for each ablation technique. The power and energy required for LA were 3 W and 1200 J, respectively, while the power and time required for RFA and MWA were 30 W and 120 s, respectively.

#### Postoperative assessment

**Fig 2:**
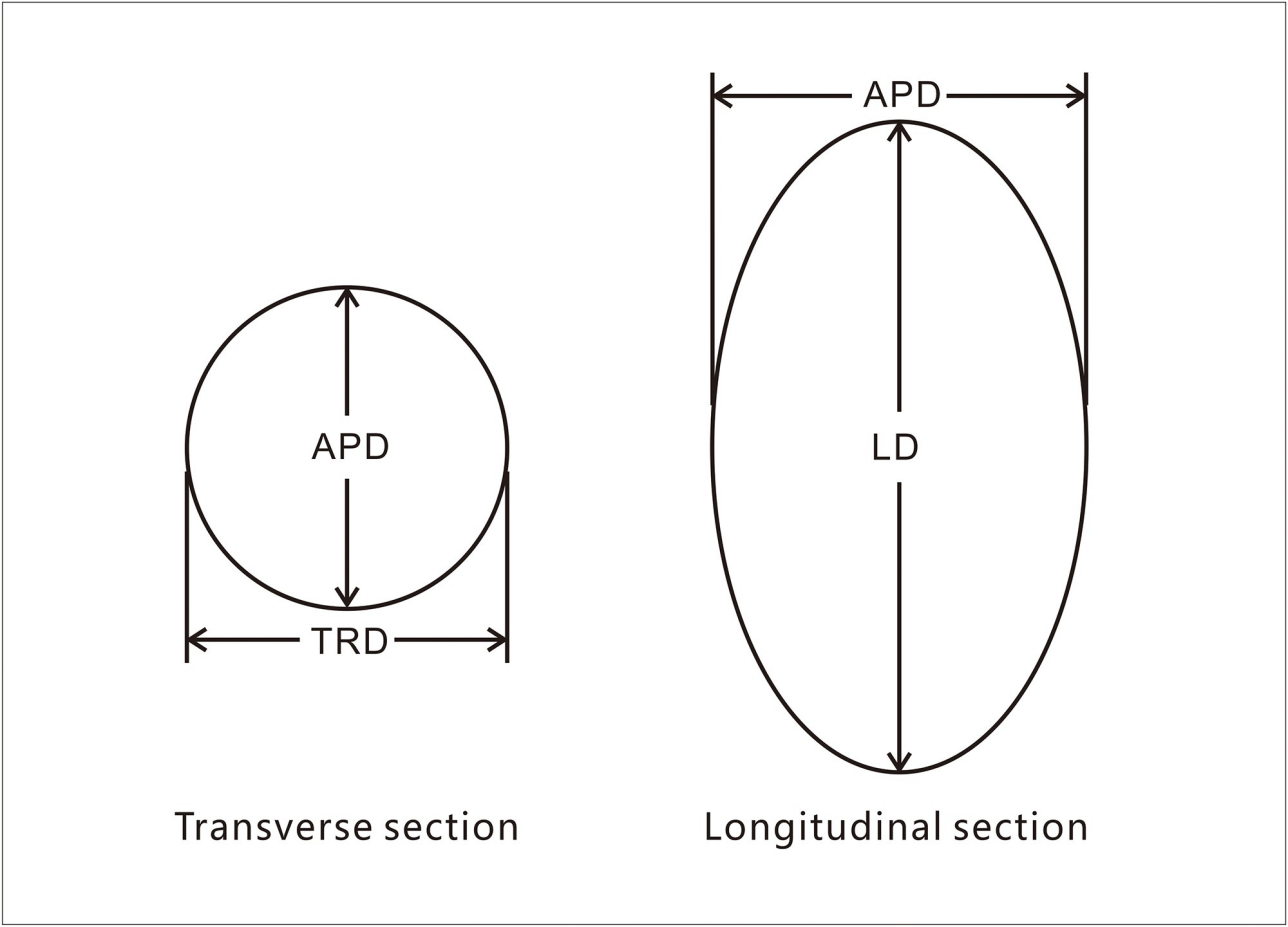
The TRD, APD, and LD were measured in the transverse and longitudinal sections.

The animals were kept warm with quilts during the waking process after anaesthesia. To minimize the influences of hyperechoic lesions induced by thermal outgassing during ablation, transrectal contrast-enhanced ultrasonography (CEUS) was performed to image the ablation lesion approximately 10 min after the ablation procedure [14]. In brief, the instrument was set to the CEUS imaging mode, a bolus injection of SonoVue was administered through the indwelling needle at the forearm vein, and then 5 ml of normal saline was used to flush the catheter. The CEUS image of each lesion was observed, and the transverse diameter (TRD) and anteroposterior diameter (APD) were measured on the transverse section of the lesion, while the longitudinal diameter (LD) was measured on the longitudinal section (Fig 2). The ratio (R), which represents the shape of the lesion, was calculated according to the following formula: R = (TRD + APD) / 2 × LD. The R value close to 1.0 indicates a more spherical shape [17,18]. The volume (V) of the lesion was calculated as follows: V = 1/6 × π × (TRD × APD × LD).

#### Postoperative care

Breathing and heart rate were closely monitored for each canine for 6 h after ablation. The canines were offered food and water 12 h after ablation. To prevent infection, each canine was intramuscularly administered 80 mg/kg gentamicin (Sinopharm Chemical Reagent Co., Ltd.) for one week following ablation. In addition, we carefully monitored the signs of haematuria, haematochezia, and pain in the canines for one month after ablation. Pain was assessed by the five assessment methods of Morton and Griffiths, which included weight, symptoms, appearance, innate behaviours and reaction to stimuli [19]. At the end of the experiment, the animals were sacrificed by injection of pentobarbital sodium (30 mg/kg).

### Statistical analysis

SPSS 22.0 software (IBM, Armonk, NY, USA) was used to perform statistical analysis. Quantitative data are described as the means ± standard deviations (SDs). Student’s t-test was utilized to compare the sizes of the lesions, while the nonparametric rank-sum test was used for comparisons among different groups. *P* < 0.05 was considered statistically significant.

## Results

**Fig 3:**
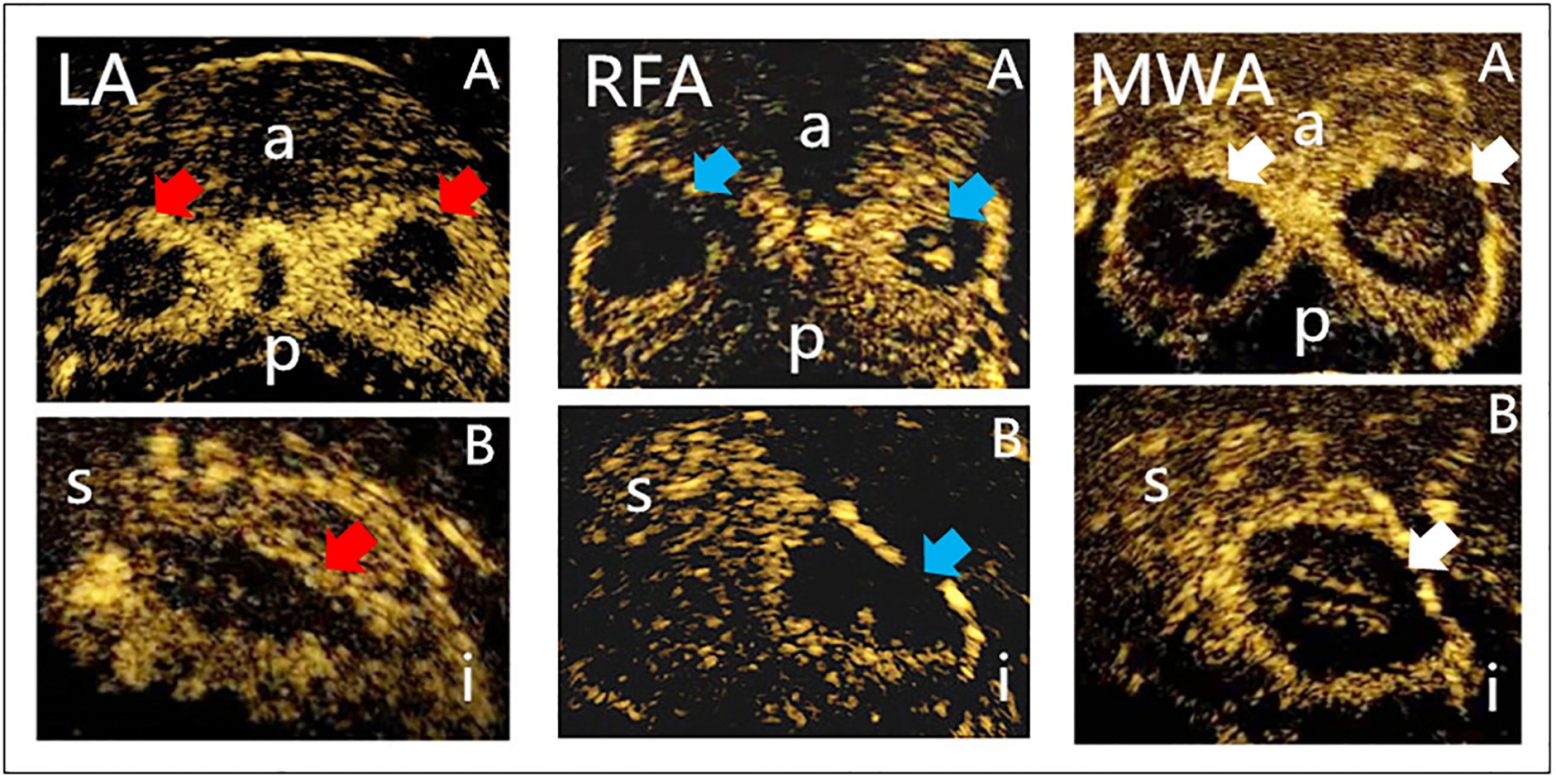
Transverse (A) and longitudinal (B) section images were observed via CEUS after LA, RFA, and MWA (red arrow, LA lesion; blue arrow, RFA lesion; white arrow, MWA lesion; a, anterior; p, posterior; s, superior; i, inferior).

Conventional TRUS revealed that the ablation lesions showed gradually expanding hyperechoic regions during the ablation process, with no distinguishable border between the lesion and normal tissue. The acoustic shadow appeared behind hyperechoic regions during ablation and disappeared after ablation. The ablation lesions were all clearly identified as an area without contrast agent and had clear boundaries and regular shapes in the CEUS images (Fig 3).

The common parameters applied in clinical practice (LA: 3 W/1200 J, RFA and MWA: 30 W/120 s) were used to ablate the tissues, and the mean R values were 0.89 ± 0.02, 0.72 ± 0.01, and 0.65 ± 0.03 for FRA, MWA, and LA, respectively, which were significantly different (*P* = 0.027). The mean volumes of the ablation lesions created via MWA (30 W/120 s) were the highest (2.17 ± 0.10 ml), followed by those of the LA (3 W/1200 J: 1.51 ± 0.20 ml) and RFA lesions (30 W/120 s: 0.79 ± 0.07 ml). The volumes of the ablation lesions were also significantly different among the three techniques (*P* = 0.001) (Table 1).

**Table 1:** Comparing the shape (R) and volume of ablation lesions created via LA, RFA, and MWA.

Ablation Techniques	LA	RFA	MWA
Power (W)	3	30	30
Time (s)	-	120	120
Power (J)	1200	-	-
TRD (mm)	12.23 ± 0.90	11.67 ± 0.59	15.70 ± 0.62
LD (mm)	18.93 ± 0.45	12.13 ± 0.32	20.73 ± 1.38
APD (mm)	12.00 ± 0.50	10.73 ± 0.25	13.83 ± 0.25
R	0.65 ± 0.03	0.89 ± 0.02	0.72 ± 0.01
V (ml)	1.51 ± 0.20	0.79 ± 0.07	2.17 ± 0.10

TRD: Transverse Diameter, APD: Anteroposterior Diameter, LD: Longitudinal Diameter, V: Volume.

## Discussion

The prostate is located at the centre of the pelvic cavity, surrounding the outer wall of the urethra, and is adjacent to a number of vital organs. In both BPH and focal PCa, it is essential for local thermal therapy to completely ablate the target while avoiding damage to surrounding organs. Therefore, local “shape-appropriate” ablation of the prostate may have positive therapeutic consequences for the patient. In the current study, LA, RFA and MWA with parameters corresponding to those in clinical practice [11,13,16] were performed in canine prostates. The volumes and shapes of the lesions were compared to provide evidence for the selection of “shape-appropriate” ablation techniques for prostate diseases.

The findings of this study showed that LA, RFA, and MWA could be used for local ablation in the prostate. During the process of LA, RFA and MWA, TRUS examination showed that the hyperechoic regions accompanied by an acoustic tail or acoustic shadow were gradually expanded. These changes were caused by the diffusion of bubbles and water in the tissues induced by the high temperature during ablation. Some studies have reported the application of hyperechoic regions for distinguishing ablation areas [20]. However, due to irregular shapes, unclear boundaries and the influence of the gas on acoustic wave transmission, some regions might be missed. In addition, the present study revealed that the hyperechoic region was transmitted to distant areas along with the interstitial space. Therefore, further studies are required to verify whether the hyperechoic regions could be used to precisely predict the ablation areas. Previous studies demonstrated the feasibility of transrectal CEUS and showed a high consistency between transrectal CEUS and pathological results in assessing the necrotic area [12,21]. In the current study, transrectal CEUS was used to assess the characteristics of the ablation lesions. The results demonstrated that the ablation lesions were areas without contrast agent filling. In addition, the boundaries of the lesions were clear, and the shapes of the lesions were regular, which were consistent with previous literature reports [12,21]. These results illustrated that it is feasible to evaluate the size of the ablation lesion with transrectal CEUS in a timely manner after ablation. However, further studies need to be conducted to indicate whether transrectal CEUS could precisely evaluate ablation lesions in humans. The shapes of the ablation lesions created via the different techniques had unique characteristics. The ablation lesions were mainly round on the transverse sections of the transrectal CEUS images. However, on the longitudinal section images, the ablation lesions created by LA were “drop-shaped”, while the ablation lesions created by RFA and MWA were more spherical. In addition, the R value, which reflects changes in the three-dimensional shape, were 0.89 ± 0.02, 0.72 ± 0.01, and 0.65 ± 0.03 for lesions created with RFA, MWA, and LA, respectively, showing distinct variability. These findings demonstrated that the three-dimensional shapes of the ablation lesions created by RFA and LA were spherical and oval, respectively, and the shapes of ablation lesions created by MWA were somewhere between a sphere and an oval.

The present study also compared the volumes of the ablation lesions created by the clinically commonly used parameters of the three ablation techniques. The results revealed that the volumes of the MWA lesions were the highest, followed by those of the LA and RFA lesions. The ablation mechanisms of the three techniques were different, and the power settings used in this study were not the same; therefore, the results lack comparability to a certain degree. However, the parameters of the three techniques used in the present study were akin to those used in clinical practice. We speculated that the evident differences in the lesion volumes could be the result of the following factors. MWA is not influenced by current conduction and carbonization of the tissues, so a large ablation lesion is created over a short period. However, during the RFA process, the carbonization induced by the high temperature hinders the radiofrequency current propagation and limits the range of ablation [22]. Although LA is not influenced by resistance, the carbonization and cavitation produced under the high temperatures at the centre of the target influences the penetrating capacity of the laser [23]. These findings are consistent with previous findings about ablation in thyroid tissues [15].

The results regarding the volumes and shapes of the ablation lesions created by the three techniques in canine prostates are of great significance for future applications in clinical practice. The ablation lesions created via RFA had the lowest volumes, and their shapes were more spherical compared to those of the other two techniques, demonstrating that RFA could be used for the treatment of relatively small lesions or tumours adjacent to vital structures and organs, such as the urethral canal and rectum. The ablation lesions created via MWA were the largest in size and spherical in shape, which could be advantageous for the ablation of tumours with relatively large sizes. The sizes of the ablation lesions created via LA were between those created via RFA and those created via MWA, and their shapes were more oval, suggesting that this method is highly appropriate for the ablation of BPH. According to a previous article [24], LA lesions showed a “narrow-stripe shape” with a single laser fibre, so LA could possibly be used to reduce urethral distortions and ablate “stripe-shaped prostate tumours” closing to the urethral canal. However, further studies are required to indicate whether these techniques could be effective in clinical practice.

In this animal experiment, we did not observe the signs of haematuria, haematochezia or pain within one month after ablation. Although the focal ablation therapy was minimally invasive, there were some complications during and after ablation process. During the ablation process, adjacent organs and structures, including the urethra, bladder and rectum, could be affected and injured. Urinary retention, urinary incontinence, recto-urethral fistula, urethral strictures and erectile dysfunction may occur after ablation in short or long period [25,26]. According to the focal LA of prostate cancer in phase I and II trials, the most common complications include haematuria, perineal ecchymosis or abrasion, glans paraesthesia, acute urinary incontinence and sexual dysfunction [27,28]. The clinical studies on transperineal RFA of the prostate were retrospective and had a small sample size and short follow-up. The most common complications included haematuria, bladder spasms, dysuria, urinary irritation symptoms, local haematoma, and so on [29,30]. Literature on the application of MWA mainly focuses on the transurethral approach, which is not comparable to the perineal puncture approach in this study. These complications may be one main limitation in the use of focal ablation therapy in prostate diseases. Therefore, focal ablation therapy in prostate diseases must be used to personalize treatment. The most appropriate ablation technique and parameters should be selected in a patient-centred manner and according to the target location, number and size of the tumours, and wishes for functional preservation.

There were several other limitations to the present study. First, the results of ablation lesion characteristics under CEUS were not compared with histopathology. Second, only the parameters commonly used in clinical practice were performed in this study to explore the characteristics of ablation lesions, but other parameters were not investigated. Third, ablation was only performed in the prostates of male adult beagles with a mating history. However, the characteristics of ablation lesions in human prostate or tumour tissues might be different. Finally, the characteristics of the lesions created with the same ablation technique might vary due to the use of different ablation devices. Further studies need to be conducted to determine whether these findings could be applied in clinical practice.

## Supporting information

S1 File The APPIVE guideline checklist for PLOS One. (DOC)
