# Supplementary material for: A pilot study of the shapes of ablation lesions in the canine prostate by laser, radiofrequency and microwave and their clinical significance
Source: PLoS One. 2020 Apr 9;15(4):e0223229. doi: 10.1371/journal.pone.0223229 (PMC7145095; doi:10.1371/journal.pone.0223229)
Supplement: S1 File — (DOC) [file pone.0223229.s001.doc]

A pilot study of the ablation lesions’ shape in canine prostate by laser, radiofrequency and microwave and the clinical significances

**Abstract** **Background**: Laser ablation (LA), radiofrequency ablation (RFA), and microwave ablation (MWA) are thermal ablation techniques, which can induce high temperature within the tissues, and lead to irreversible damages to tissues. To date, no studies have reported which of the three ablation techniques should be selected for BPH and PCa. **Objective:** To explore the ablation lesions’ shapes in canine prostate underwent laser ablation (LA), radiofrequency ablation (RFA) and microwave ablation (MWA).and clinical significances. **Methods**: The six male Beagle dogs were randomly assigned to LA, RFA, and MWA groups, respectively. The ablations were conducted with the doses commonly applied in clinical practice (LA: 3 W/1200 J; RFA and MWA: 30 W/120 s) for one region each lobe of the prostate, which totally resulted in 12 ablation lesions. The energy transmitter was inserted via the perineum under the guidance of transrectal ultrasound (TRUS) and the ablation process was observed dynamically. After ablation, the efficacy was assessed by contrast-enhanced ultrasonography (CEUS)，and the transverse diameter (TRD), anteroposterior diameter (APD) and longitudinal diameter (TRD) of each ablation lesion were measured. Then, the volume (V) was calculated according to the following formulae, which was used to show the shape of the lesion. **Results:** The shapes and sizes of the lesions underwent the three techniques were different under the clinically commonly used doses. The R values, reflecting the changes in shapes of lesions, were 0.89±0.02, 0.72±0.01, and 0.65±0.03 for RFA, MWA and LA, respectively, which were significantly different (*P* =0.027). The volumes of the lesions underwent the three ablation techniques significantly different as well (P=0.001), which were 2.17±0.10, 1.51±0.20, and 0.79±0.07 ml for MWA, LA and RFA, respectively. **Conclusion**: The above-mentioned three techniques could be used for the ablation of the prostate using the clinically commonly used doses. The shape and volume of ablation which created by the three ablation techniques under the common doses in clinical were different. The shapes of the lesions which created by the three techniques were different evidently, with the RFA and LA were more spherical and oval, respectively, while the MWA was between spherical and oval. The lesions received MWA and RFA had the largest and smallest sizes, respectively, while the sizes of lesions underwent LA was in the middle. These findings may assist physicians to select an appropriate ablation technique for the treatment of prostate disease.

**Introduction**

Benign prostatic hyperplasia (BPH) is a disease frequently found in middle-aged and old men, which could induce lower urinary tract symptoms (LUTS), and therefore, the LUTS severely affect patients’ quality of life [1]. Drug therapies and surgical treatments have been used for the treatment; however, both approaches accompany with a number of side-effects [2]. A study on epidemiology showed that the incidence and mortality of prostate cancer (PCa) are remarkable in males [3]. The conventional therapeutic strategies include operation, radiotherapy, and endocrine therapy [4], however, these treatments all have certain influences on sexual functions, urination, and defecation [5]. Local minimally invasive treatment possesses several great advantages, including being minimally invasive, repeatable, as well as associating with fewer side-effects. Therefore, increasing attention have been paid on this method, which has been extensively applied in clinical practices [6,7]. Laser ablation (LA), radiofrequency ablation (RFA), and microwave ablation (MWA) are thermal ablation techniques, which could induce high temperature within the tissues, and may lead to irreversible damages to tissues. LA has already been conducted in a variety of countries for the treatment of prostate disease [8,9], and the effectiveness and safety profiles have been demonstrated. The FRA and MWA were conducted in animal experiments as well. Pathological examinations have demonstrated that RFA and MWA could induce coagulative necrosis. The high-power MWA (45 W/180 s) results in the penetration of ablation through the prostate, suggesting that appropriate power and time should be taken for the treatment into consideration [10,11]. To date, no studies have reported which of the three ablation techniques should be selected for BPH and PCa. In the present study, animal experiments were conducted to explore the characteristics related to shapes and clinical significances of the lesions of canine prostate underwent the three types of ablation techniques, using the most frequent clinically used doses. Our findings may provide assist physicians to select an appropriate ablation technique for thermal ablation of prostate diseases in clinical practice.

**Methods**

*Ethical statement:* The study protocol was approved by the Ethics Committee of the Center for Drug Safety Evaluation of Zhengzhou University (Zhengzhou, China). The ethics committee of this organization contains animal welfare experts. The ethics number of this animal study was ZZUCDSER012.

*Study design:* The ablations were conducted with the doses commonly applied in clinical practice (LA: 3 W/1200 J; RFA and MWA: 30 W/120 s) for one region in the left and right lobe of the prostate, which totally resulted in 12 lesions. To explore the difference in shapes and volume in three groups.

*Experiment procedures:* The experiment was operated in the animal laboratory at Henan provincial people’s hospital. First, Under general anesthesia after fasting for 12 h, the animals were fixed on the operating table. Secondly, An energy transmitter was inserted via the perineum under the guidance of transrectal ultrasound (TRUS) and the ablation process was observed dynamically. Thirdly, the lesions in the prostate were assessed by transrectal contrast-enhanced ultrasonography (CEUS).

*Experiment animals:* 6 adult male Beagle dogs with mating history were purchased from Dilepu Biomedical Co., Ltd. (Xian, China); their mean age was 5.2±0.8 years old, and their body weight was 14.2±2.2kg. The dogs were kept in the Center for Drug Safety Evaluation of Zhengzhou University (Zhengzhou, China).

*Housing and husbandry:* The animals were individually housed in stainless steel cages (L 1000 x W 1000 x H 2100 mm) before and after ablation. The environment in which dogs live is set to 23 ± 3 ℃ of temperature, 55 ±15 % of relative humidity, 10~20 times/hr of ventilation frequency, 12 h of lighting duration (lighting up at 8 a.m. ~ lighting out at 8 p.m.) and 150~300 Lux of luminous intensity. Each dog was offered a daily ration of 300 g of solid food (Beijing Keao Xieli Feed Co., Ltd.). The uneaten food was collected on the following morning. Water was disinfected by ultraviolet sterilizer and ultrafiltration and made available ad libitum using an automatic water supplier.

*Simple size:* There were six beagle dogs in this experiment. The beagle's prostate anatomy is similar to that of humans. However, In view of the prices of beagle dogs and two lobes in each dog prostate, there were two animals and four experiment sites in each group.

*Allocating animals to experimental groups：*The animals were ranked according to their weight, and were randomly assigned to LA, RFA and MWA groups by random number table. *Experimental outcomes:* the primary outcomes: To explore the shapes and clinical significances of the lesions of canine prostate underwent LA, RFA, and MWA. The complications about animals during the operation and after surgery were observed and recorded. The animals are kept warm by quilts during the waking process after anesthesia. The breathing and heartbeat were closely monitored for each dog for 6 h after ablation. The dogs were offered food and water after ablation 12 h. To prevent infection, each dog was intramuscularly administered 80mg/kg gentamicin (Sinopharm Chemical Reagent Co., Ltd.) for one week following the end of ablation Besides, we pay attention to the physical sign of hematuria, hematochezia and pain about dogs. After the completion of follow up observation, the animals were sacrificed by injection pentobarbital sodium (30mg/Kg ) at the end of experiment.

The secondary experiment outcomes: To observe dynamic changes of ablation site in a mouth.

*Statistical methods:* In this present study, SPSS 22.0 software (IBM, Armonk, NY, USA) was used to perform statistical analysis. Quantitative data were described as mean ± standard division (SD). Student’s t-test was utilized to compare the size of lesions, while the non-parametric rank-sum test was used for making comparisons among different groups. P<0.05 was considered statistically significant.

**Results**

*Baseline data:*

| Group | Weigh(kg) | Age(y) |
| --- | --- | --- |
| LA | 16.2 | 6 |
| LA | 14.4 | 4.2 |
| RFA | 12.1 | 5.2 |
| RFA | 14.1 | 5.2 |
| MWA | 12 | 4.4 |
| MWA | 17.75 | 6.3 |

*Outcomes and estimation :*

| Ablation Method | LA | RFA | MWA |
| --- | --- | --- | --- |
| Power (w) | 3 | 30 | 30 |
| Time (s) | - | 120 | 120 |
| Power (J) | 1200 | - | - |
| TRD (mm) | 12.23±0.90 | 11.67±0.59 | 15.70±0.62 |
| LD (mm) | 18.93±0.45 | 12.13±0.32 | 20.73±1.38 |
| APD (mm) | 12.00±0.50 | 10.73±0.25 | 13.83±0.25 |
| R | 0.65±0.03 | 0.89±0.02 | 0.72±0.01 |
| V (ml) | 1.51±0.20 | 0.79±0.07 | 2.17±0.10 |

**Discussion**

We know from experiments that the size of the prostate with adult male beagle dogs was suitable for prostatic ablation experiments. Besides, during the ablation process, the use of intraoperative intravenous anesthesia reduce animal suffering and ensure the personal safety of experimenters. The anesthetic drugs and contrast agents are calculated by reference to the articles, which was safe for animals.

During the operation, there were no complications with the dogs. The findings of this study showed that LA, RFA, and MWA could be used for the local ablation of the prostate.

The results related to the sizes and shapes of the prostates of the dogs that underwent the three ablation techniques are of great significance for future application in clinical practice. The lesions underwent RFA had the lowest size, and their shape was more spherical, demonstrating that the mentioned method could be used for the treatment of lesions with a relatively small size, or tumors surrounded by vital organs, such as urethral canal, rectum, and seminal vesicle. The lesions underwent MWA had the highest size, while their shape was spherical, which could be advantageous for the ablation of tumors with relatively large size. The size of lesions received LA was between those underwent RFA and MWA, and their shape was more oval, suggesting that the proposed method is highly appropriate for the ablation of BPH. As lesions showed a “narrow-stripe shape” [27], the proposed method could possibly reduce urethra distortion and could be therefore used for the ablation of “stripe-shaped prostate cancer” close to the urethral canal. However, further studies are required to indicate whether these techniques could be effectively helpful in clinical practice.

There were several limitations in the present study. Firstly, only the clinically commonly used doses were taken in this study into account to explore the size and shape of lesions, while the other parameters were not investigated. Secondly, the only prostate of normal dogs was ablated in this study, while the ablation area in tumor tissues or human prostate might be different. Finally, for the same ablation technique, the characteristics of lesions might vary due to the use of different types of ablation devices. Further researches need to be conducted to indicate whether the findings could be applied in clinical practice.

Funding Statement 1: This work was supported by the talent project fund of Henan provincial people's hospital ( ZC23456090 ) which belong to prof. Zhang Lianzhong. Prof. Zhang Lianzhong is the corresponding author, who taking part in the designing study and the revising paper.

Funding Statement 2: This work was supported by the talent project fund of Henan provincial people's hospital ( ZC23456090). The funders had no role in study design, data collection, and analysis, decision to publish, or preparation of the manuscript.
